# Multidisciplinary intervention to improve medication safety in nursing home residents: protocol of a cluster randomised controlled trial (HIOPP-3-iTBX study)

**DOI:** 10.1186/s12877-019-1027-0

**Published:** 2019-01-25

**Authors:** Olaf Krause, Birgitt Wiese, Ina-Merle Doyle, Claudia Kirsch, Petra Thürmann, Stefan Wilm, Lisa Sparenberg, Regina Stolz, Antje Freytag, Jutta Bleidorn, Ulrike Junius-Walker, for the HIOPP-3-iTBX study group, Simone Bernard, Stefanie Kortekamp, Angela Fuchs, Achim Mortsiefer, Anja Wollny, Attila Altiner, Hannah Haumann, Stefanie Joos, Nils Schneider, Thomas G. Grobe, Christian Günster

**Affiliations:** 1 Institute for General Practice, Hannover Medical School, Carl-Neuberg-Straße 1, 30625 Hannover, Germany; 2 Philipp Klee Institute for Clinical Pharmacology, University of Witten/Herdecke, Heusnerstraße 40, 42283 Wuppertal, Germany; 3 Institute for General Practice, Heinrich-Heine University Düsseldorf, Werdener Straße 4, 40227 Düsseldorf, Germany; 4 Institute for General Practice, University Medical Center Rostock, Doberaner Straße 142, 18057 Rostock, Germany; 5 Institute for General Practice and Interprofessional Care, University Hospital and Faculty Tübingen, Osianderstraße 5, 72076 Tübingen, Germany; 6 Institute of General Practice and Family Medicine, Jena University Hospital, Bachstraße 18, 07743 Jena, Germany

**Keywords:** Nursing homes, Polypharmacy, Medication review, Medication therapy management, General practitioners, Pharmacists, Nursing staff, Interprofessional, Change management

## Abstract

**Background:**

Medication safety is an important health issue for nursing home residents (NHR). They usually experience polypharmacy and often take potentially inappropriate medications (PIM) and antipsychotics. This, coupled with a frail health state, makes NHR particularly vulnerable to adverse drug events (ADE). The value of systematic medication reviews and interprofessional co-operation for improving medication quality in NHR has been recognized. Yet the evidence of a positive effect on NHR’ health and wellbeing is inconclusive at this stage. This study investigates the effects of pharmacists’ medication reviews linked with measures to strengthen interprofessional co-operation on NHR’ medication quality, health status and health care use.

**Methods:**

Pragmatic cluster randomised controlled trial in nursing homes in four regions of Germany. A total of 760 NHR will be recruited. Inclusion: NHR aged 65 years and over with an estimated life expectancy of at least six months. Intervention with four elements: i) introduction of a pharmacist’s medication review combined with a communication pathway to the prescribing general practitioners (GPs) and nursing home staff, ii) facilitation of change in the interprofessional cooperation, iii) educational training and iv) a “toolbox” to facilitate implementation in daily practice. Analysis: primary outcome - proportion of residents receiving PIM and ≥ 2 antipsychotics at six months follow-up. Secondary outcomes - cognitive function, falls, quality of life, medical emergency contacts, hospital admissions, and health care costs.

**Discussion:**

The trial assesses the effects of a structured interprofessional medication management for NHR in Germany. It follows the participatory action research approach and closely involves the three professional groups (nursing staff, GPs, pharmacists) engaged in the medication management. A handbook based on the experiences of the trial in nursing homes will be produced for a rollout into routine practice in Germany.

**Trial registration:**

Registered in the German register of clinical studies (DRKS, study ID DRKS00013588, primary register) and in the WHO International Clinical Trials Registry Platform (secondary register), both on 25th January 2018.

## Background

The medication management of nursing home residents (NHR) is complex and can be challenging for all involved professions [1]. In Germany, doctors prescribe, community pharmacists dispense and nurses administer drugs to NHR. Due to the continuous contact with the NHR, nurses are in the best position to observe their health state. They may, however, lack knowledge about geriatric pharmacotherapy and thus cannot decide which of the changes observed in health status might be associated with drug therapy. In Germany, only doctors are authorised to prescribe, but often they do not have enough time for thorough medication reviews. On the other hand, pharmacists have in-depth knowledge on medications’ effects and interactions, however, reviewing medications is generally not part of their task portfolio and is not remunerated. Hence cooperation of the three professions is a promising approach for a safe and efficient medication management [2]. Systematic procedures on how to co-operate are not in place [3], and the professional groups involved in medication management (GPs, nursing staff and pharmacists) have been shown to poorly communicate and undervalue each other’s competencies on this matter [4].

There are strong arguments in favour of a careful medication management for NHR. Firstly, NHR are commonly exposed to polypharmacy. In a study of 21 German nursing homes, 70% of NHR received 5 or more prescribed medications [5]. Polypharmacy increases the risk of drug-interactions, and adverse drug events (ADE) such as falls [6]. Secondly, the majority of NHR has a frail health state and/or suffers from dementia. Frailty poses an additional risk of experiencing ADE resulting in earlier incident disability and mortality [7] . Thirdly, a great number of NHR are exposed to potentially inappropriate medications (PIM) [8]. Studies have reported that approximately 40% of residents receive PIM [9, 10] compared to 20–25% of patients living in the community [10]. Indeed, 20% of NHR take medication that is contraindicated or incorrectly dosed for their level of renal function [11]. Another safety issue is the administration of antipsychotic drugs [8, 12, 13]. Studies from Germany and Austria indicate that more than half of all NHR receive such drugs, the necessity of which is often proposed by nursing home staff [14].The inherent risks are over-sedation, falls, fractures [15], arrhythmias, cerebrovascular events and even premature deaths [12, 16]. These factors together present a comprehensive risk that has been associated with a clustering of adverse drug events and premature deaths for NHR [12].

Internationally, studies relating to optimising medication management in nursing homes are increasingly available. They are designed using different interventions such as pharmacist led medication reviews, improved interprofessional collaboration, training of staff or providing IT-support for medication reviews. Often these studies apply a mixture of strategies. Yet the emphasis most often lies on the pharmacist review. Several systematic reviews have attempted to pool the heterogeneous findings on the prescribing quality and NHR’ health [17–19]. The most comprehensive review by Alldred et al. includes 12 studies [19]. The investigators conclude that no firm conclusions on the effects can be drawn. However, there is an indication that medication management interventions may lead to a better quality of prescribing whereas little evidence can be found so far on health improvements [19]. Other recent reviews do not focus on nursing homes [20] or not solely aim to optimising medication for NHR [21].

In Germany, interventional studies (AMTS Ampel I and II) [2, 22] report a decrease in avoidable ADE using a multicomponent intervention including pharmacists’ advice. Yet to our knowledge a randomised trial aiming to optimise the medication management for NHR has not been undertaken.

The HIOPP-3-iTBX study stands for “General Practitioners’ Initiative to optimise Medication safety for Nursing Home Residents” – using an “interprofessional toolbox” with focus on “three main stakeholders” namely GPs, nursing staff and pharmacists. The intervention involves a pharmacist led medication review, training of all professional groups and support in a change management process using participatory action research (PAR). PAR actively involves the main stakeholders in the change and strengthens the awareness of interactions between them. The method also strengthens self-emerging context-specific solutions in nursing homes [23].

The aim of the transregional cluster randomised study is to investigate the effect of a multicomponent medication safety intervention on the prescribing quality and NHR’ health. The primary outcome is the rate of NHR receiving potentially inappropriate medications (PIM) and/or ≥ 2 antipsychotic drugs; the secondary outcomes relate to the number of falls, emergency medical contacts, hospitalisation, health costs and quality of life and cognition of NHR. We hypothesise that our interprofessional intervention will lead to an improved and more efficient medication management and to a sustainable optimisation of the medication process involving nurses, GPs and pharmacists.

## Methods

### Design and setting of the study

**Fig. 1:**
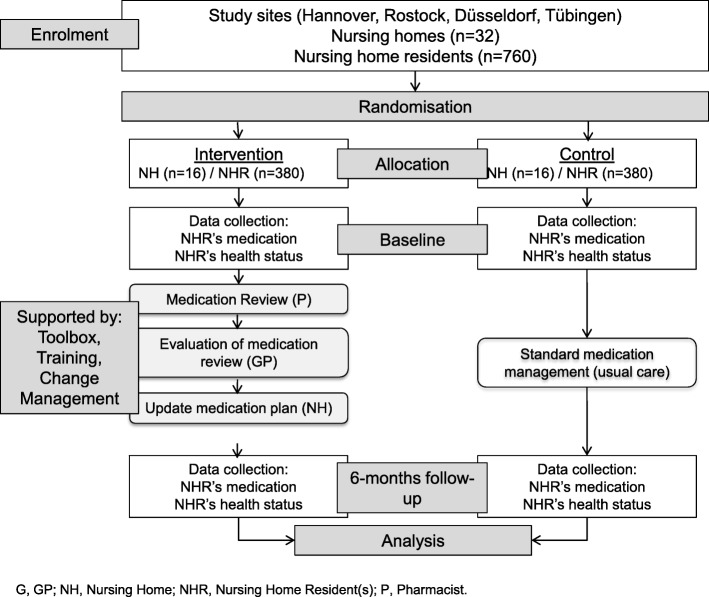
Flow chart with an overview of study steps

The multicentre cluster randomised controlled trial (cRCT) is conducted in four geographical regions in Germany (Hannover, Rostock, Düsseldorf and Tübingen), of which two have a wide rural periphery and two are more urban based. Clustering takes place at the level of nursing homes. Together, the four study centres will include a total of 760 NHR at baseline (Fig. 1).

### Participants and materials

Study participants are NHR who are at least 65 years of age and living in long-term residential care facilities. Exclusion criteria are having a life expectancy of less than 6 months based on nurses’ judgement. In case of doubt or when a nurse’s judgement is not available, the NHR’ GPs are asked for their judgement. The eligibility criteria for nursing homes, doctors and pharmacists are described in Table 1.

**Table 1:** Inclusion and exclusion criteria

	Inclusion criteria	Exclusion criteria
Nursing home	Size at least 30 residents, and nursing home status in accordance with German statutory social law.	Specialised nursing homes (nursing homes for ventilated patients, psychiatric homes, homes for alcohol or drug addicted patients)
Pharmacist	Serving a nursing home participating in the study, willing to be trained in performing the medication review if randomised to the intervention group.	Pharmacists who serve intervention and control group nursing homes at the same time
GP	Serving a nursing home participating in the study, willing to provide participants’ health data (current diagnoses, laboratory parameters), willing to participate in a special training if randomised to the intervention group	Currently participating in other ongoing activities about medication of nursing home residents, GPs who concurrently serve NHR in intervention and control homes
NHR	At least 65 years old and in long-term residential care	Residents with a life expectancy less than six months based on nurses’ judgement and, in case of doubt, additionally based on the GPs’ judgement, patients in vigilant coma

### Recruitment strategy

The recruitment procedure is standardised and follows several steps. First, all nursing homes covering the four areas are informed about the study by mail (September 2017). Nursing homes expressing an interest are contacted by phone or personal visit in order to describe the study in more detail, to assess whether eligibility criteria are met and whether the institution is willing to participate (see Table 1).

In a second stage, the pharmacists supplying the nursing homes and all GPs responsible for the residents of the selected nursing homes are approached by mail or fax followed by a phone call in case of a non-response (November 2017 to February 2018). One pharmacy usually supplies one nursing home. By contrast, typically several GPs share the care of NHR in one facility so that several GPs might participate per nursing home residency. If both, consent and eligibility criteria are met for pharmacists and GPs, recruitment of that nursing home can proceed to the third stage.

In the third and final step, NHR cared for by a participating GP are recruited. For ethical and data protection reasons, the nursing home staff initially approaches their residents with information about the study. If the NHR consents to be contacted by our study personnel, they receive detailed written and verbal information and clarification of any queries. If a resident has a custodian, this person is contacted by the study personnel or by the nursing home manager. For the custodians we use an adapted version of the study information and the consent form. In case of custodianship, both resident and custodian have to give their written consent or else the resident cannot be recruited. In cases where only one party gives written consent, a participation in the study is not possible. An exception are cases where residents cannot give written consent, e.g. due to cognitive or physical impairment, in which the sole written consent of the custodian is sufficient.

The consent form includes two parts: i) consent to the potential intervention and the sharing of the necessary personal and medical data and ii) consent to the cognitive assessment Mini-Mental State Examination (MMSE) [24]. It is possible to participate in the study without performing the MMSE but not without performing part i). After written informed consent has been obtained, study nurses input baseline data such as current diagnoses, laboratory parameters (obtained from the GPs) and symptoms as well as medication prescriptions into a trial database (SecuTrial®). Pharmacists can commence the medication review once the study nurse tells them that all necessary data is complete and they have received log-in data for secuTrial®.

### Intervention

Following baseline assessment (t0), the intervention will be conducted and its effects measured at 6 months follow-up (t1) (Fig. 1). The intervention consists of four elements.

Firstly, the community pharmacists in charge of the nursing homes receive a special two-day training (ATHINA = Arzneimitteltherapiesicherheit in Apotheken i.e. medication therapy safety in pharmacies) [25] in performing an IT-based medication review and a further one-day training in geriatric pharmacotherapy especially designed for the study (HIOPP-3 training) based on programmes developed for previous studies (AMTS AMPEL I and II; [2, 22]). After the completion of baseline documentation by study nurses the pharmacists perform a structured medication review. Pharmacists are trained to consider 13 potential medication problems for their medication review:

- no indication for a certain drug based on available diagnoses
- negative benefit-risk profile (according to pharmacist’s judgement)
- potential relevant drug interaction
- potential relevant drug-disease interaction
- duplicate prescription
- PIM in elderly (PRISCUS list) [26]
- potentially relevant side effects
- application/usage inappropriate
- possible underdosing
- possible overdosing
- contraindication
- assess duration of treatment
- other reasons

The results and advice will be documented in secuTrial® and transferred to a special fax template (“medication check fax”), to be sent to the responsible GPs. The GPs in turn assess the pharmacists’ suggestions and amend their medication prescriptions, if they deem the suggested changes appropriate. Additional prescribing specialist doctors (e.g. psychiatrist) may also be informed of medication changes, either via the GP or the nursing home.

Secondly, change management principles are introduced to enhance the involved professions’ understanding of roles and interprofessional communication as a vital part of the medication management [27]. Change management support is provided by means of three workshops which are conducted in the nursing home and to which all participating professional groups are invited. The first workshop is held around the time of baseline data collection. Trained study personnel and project managers inform the attendees about the study and its goals. They facilitate a strength and weaknesses analysis of the current medication management structures and processes. Input from all attendees is noted on cue cards. Following a discussion, specific goals for each nursing home are set and suitable tools and processes to facilitate the change process (see below in Table 2) are introduced to support communication and collaboration. Finally, an intervention handbook describing all elements of the intervention/tool box is handed out.

**Table 2:** Toolbox content

Name	Function
Thematic map for drug safety (German name: AMTS-AMPEL-Karte)	• clues for drug induced symptoms • uses a simple traffic light system:red: for high risk drugs yellow: weighty symptoms to look out for green: clues for the need of clinical monitoring • to be used by nursing staff and GPs
Additional information for the thematic map for safety in drug therapy	• easy explanations for drugs and symptoms • to be used by nursing staff
Treatment observation sheet (German name: Therapiebeobachtungsbogen)	• to be used by nursing staff • records ongoing/new symptoms in NHR • to look out for adverse drug events
Ward round tool	• to be used in common ward roundsof all 3 partaking professions • for medical and pharmacological issues of NHR
PRISCUS list [26]	• List of potentially inappropriate medications (PIM) for senior citizens • contains 83 PIM
Hospital discharge tool	• communication aid between nursing staff and GPs • used after discharge from hospital

A second change management workshop is held 2 months later. All stakeholders discuss how they have experienced the initiation of change and what further support or agreements are necessary to progress in improving their medication management. New tools can be introduced at this stage. Attendees are also asked to finalize an action plan detailing how they wish to cooperate in the following months and which tools they intend to use.

The final change management workshop is held at the end of the intervention period (after 6 months). Here the study personnel aim to get feedback on the perceived degree and success of change, on facilitators and barriers for a potential nation-wide roll-out into routine practice. Successes will be celebrated and agreements between stakeholders for a sustained local cooperation will be made.

All workshops are protocolled, and if consent is given, audio recorded for a comprehensive change analysis across different sites.

The third element of the intervention consists of educational trainings for each professional group on polypharmacy, PIM, antipsychotics and medication management in the nursing home setting. Training is offered according to the specific needs of each professional group. The pharmacists receive the most extensive training. GPs obtain an up to 2 hour training in geriatric pharmacotherapy and information on how to use the tools to improve interprofessional collaboration. The nursing staff receives two shorts educational sessions on “adverse drug events” and “antipsychotics” in NHR.

The final element of the intervention, the toolbox, supports the practical application of new competencies offering a variety of tools for daily use. Some of the tools have been developed and tested in previous studies [2, 22]. The partaking professions can choose voluntarily from the toolbox’ items. The content of the toolbox is shown in Table 2.

### Incentives

Pharmacists allocated to the intervention group as well as GPs receive a financial compensation per medication review. As part of the preparatory phase, the ATHINA training and HIOPP-3 training sessions are offered to all pharmacists allocated to the intervention group free of charge. Pharmacists allocated to the control group can receive the ATHINA training after study completion.

Furthermore, the toolbox and findings of our study will be made available to all interested nursing homes, GPs and pharmacists after study completion in the form of a “manual”.

### Outcomes

The primary endpoint is the rate of PIM and/or antipsychotic drugs, which will be calculated based on the current medication data. The secondary endpoints/parameters are number of falls, emergency medical contacts, hospitalisation, quality of life and cognition of NHR. Moreover, health services utilisation is assessed for a health economics evaluation. Data are retrieved from the NHR themselves by interview and supplemented by routine NHR documentation in the participating nursing home. An overview of all collected data and time points is given below (Table 3).

**Table 3:**
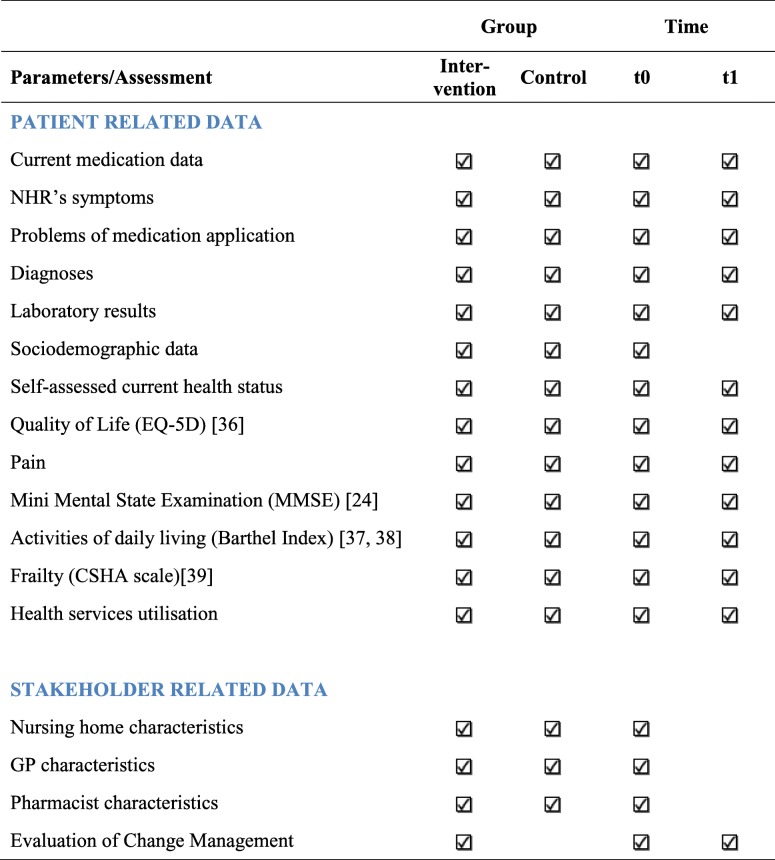
Overview of planned assessments/data collection [24, 36–39]

### Sample size

The sample size estimation is based on the assumption that a proportion of 50% of NHR receive PIM and/or ≥ 2 antipsychotics in Germany. This estimated figure is based on studies according to which 40% of NHR receive at least one PIM [10] and 20% receive two and more antipsychotics simultaneously [28]. We assume that an overlap of PIM and ≥ 2 antipsychotic use exists in 10%. We expect a meaningful reduction in the proportion of NHR from 50 to 30% receiving PIM and/or ≥ 2 antipsychotics.

An intracluster correlation (ICC) of ρ = 0.1 is assumed and a mean number of 25 residents per nursing home is targeted. Under these assumptions and with an α = 0.05 and a power of 80% a total of 632 residents are necessary to detect the expected difference (two-sided χ^2^-test). The one-year mortality rate is conservatively estimated as 30% [29]. With a six-months-follow-up we assume a drop-out-rate of 20% (NHR’ life expectancy in months: 47 for women/26 for men) [30]. Altogether 760 patients (and 32 nursing homes) are needed.

### Randomisation

The randomisation is carried out as a cluster randomisation allocating the nursing homes either to the intervention or to the control group. Randomisation is performed by an experienced biometrician at Hannover Medical School after the recruitment of patients (BW). The allocation sequence is computer generated and concealed from researchers and interviewers. The randomisation is stratified by centres to ensure a balance in the number of nursing homes and patients allocated to each treatment group within the centres.

### Blinding

Due to the complex nature of the intervention, blinding of participants, health care providers and data collectors is not possible. The randomisation is performed after the recruitment of nursing homes, pharmacists, GPs and nursing home residents.

### Data collection methods

Resident-related data are collected by the study personnel via standardised questionnaires and additionally according to the NHR’s records. Then the data is entered into an internet based electronic data capture system at two time points: t0 after obtaining written informed consent and randomization and before starting the intervention, t1 after the intervention period (month six).

### Data management

Data will be entered in the local centres via an internet based electronic data capture (EDC) system which complies with FDA requirements (21 CFR Part 11) and the guidelines for Good Clinical Practice (GCP). The data will be stored in a central database (Oracle); there is no local data storage. The data are transferred via 128 bit SSL encryption. The access to the database and webserver is controlled by two consecutive firewall systems. Data will be stored with a pseudonym. The members of the study group have access to the electronic data entry system according a detailed concept of roles and rights. An audit trail ensures an automatic protocol of all data entries, changes and deletions.

### Statistical analyses

Because of the cluster randomisation multilevel models with the nursing home as random effect (e.g. mixed model logistic regression) will be applied for the statistical analyses of primary and secondary endpoints. Possible imbalances and confounding variables will be included in the statistical models for adjustment. The data will be analysed according the intention-to-treat principle.

### Quality assurance and safety

Quality assurance consists of procedures for prevention of insufficient data quality, detection of inaccurate or incomplete data and action to improve data quality, e.g. user training sessions, automatic plausibility and integrity checks within the electronic data capture system and data error reports for the local centres. Reliability trainings and checks will be performed before starting the study with the whole staff involved in interviewing, medication review and data collection. In addition the centres will regularly receive feedback by quality reports. Additionally, clinical pharmacologists will double check a certain amount of the performed medications reviews after the intervention phase.

### Evaluation

Different parts of the study will be evaluated:

1. The awareness to the need for changing medication management and later the satisfaction with the change and the perceived benefit will be evaluated in all three workshops in a discussion (focus group type) and with additional questionnaires.
2. The acceptance and usage of the different HIOPP-3 tools will be recorded in the interim and final workshop in the nursing homes.
3. Some of the performed pharmacists’ medication reviews will be randomly “double-checked” by a peer clinical pharmacologist after the intervention period.

The public will be informed about the aims, culture, structure and processes of the medication management in the participating nursing homes. Facilitators and barriers will be mapped in order to develop a roll-out and implementation guidance for routine healthcare in Germany. The handbook will detail steps and guidelines on how to implement a collaborative medication management taking into consideration successful supportive training and management tools.

### Trial status

At the time of submission of this manuscript in January 2019, recruitment, training for all participating professional groups, baseline data collection, change management workshops and medication reviews have been concluded in more than half of the nursing homes. The intervention phase is still ongoing.

## Discussion

Up to date, systematic reviews assessing the available evidence of intervention studies in NHR that include a medication review have found positive effects on medication quality and surrogate parameters like number of drugs or medication appropriateness [17–19]. However, convincing beneficial evidence on clinical endpoints such as hospitalisation and mortality could not be established. Yet withdrawing medications without negative effects may be already regarded as a success, as Wouters et al. [31] point out. It is also possible that a number of factors relating to the study designs and their conduction might have impeded positive effects on health. Weaknesses, which have been mentioned in reviews, are underpowered studies, heterogeneous study designs, low quality studies, or possibly confined or non-sustained interventions [19–22]. Indeed, it may not be very conducive to prepare nursing home professionals for a one–off medication review without providing continuous support on how to sustainably change the way nurses, doctors and pharmacists cooperate with each other.

HIOPP-3-iTBX tries to overcome some potential obstacles, as it is a large multi-centred cluster RCT which employs a broad intervention that goes beyond a medication review. All professions primarily involved in the medication management of NHR actively participate in the intervention which uses a multi-pronged approach that acts on all those levers identified as relevant in improving medication management: knowledge, education and improved interprofessional communication and cooperation.

This is a growing research field and fortunately similar studies are on their way, which emphasise a structured approach and/or focus on multiprofessional cooperation. The German InTherAkt study is a single arm study in NHR aiming to improve the Medication Appropriateness Index via enhanced interprofessional cooperation [32]. A cRCT in German nursing homes (EPCentCare) has started aiming to investigate whether a person-centred care approach developed in UK leads to a reduction of antipsychotic prescribing [33]. The COME-ON cRCT is a complex, multifaceted intervention, including interdisciplinary case conferences, on the appropriateness of prescribing of medicines in Belgian nursing homes [34]. The SCREAM study aims to use a computerised clinical support system that analyses the NHR’ medications in terms of interactions, dose appropriateness and other clinical data to reduce the time resources for medication reviews by pharmacists and nursing home physicians [35]. Our study’s emphasis is on the combination of a standardised pharmacist-led medication review and changing interprofessional cooperation.

### Challenges

We are aware of several challenges pertaining to the conduction of the trial in the fields of recruitment, motivation of the professionals in the intervention and control groups, and drop out.

The recruitment of nursing homes is limited due to the difficulty matching interested nursing homes with responsible pharmacists and interested GPs. Once the matching has taken place, recruiting NHR is confined to only those, whose health professionals have consented. Moreover, often legal caretakers are to be involved who additionally have to consent.

We also expect difficulties in keeping nursing homes, GPs and pharmacists allocated to the control groups motivated to provide current diagnoses and laboratory parameters of their participating patients as they will not receive a compensation for this. Once community pharmacists are randomised into the control group they do not perform a medication review and might feel disappointed. We aim to overcome these obstacles by offering nursing homes, GPs and pharmacists in the control group access to intervention procedures at the end of the study.

Finally, we anticipate an extensive loss-to-follow up of the NHR due to a short life expectancy [30], or discontinuation of participating health professionals. Our sample size calculation addresses this issue allowing a drop out of 20% of NHR within the 6 months intervention phase.

### Outlook

In a nutshell, the HIOPP-3-iTBX study represents a pragmatic cluster randomised trial in nursing homes applying a pharmacist-led medication review, interprofessional training, a toolbox and change management support. In the light of an ageing population and increasing number of older people being cared for in nursing homes, the interprofessional optimisation of their drug therapy is a major health issue. If proven successful, we intend to compile guidance on implementing this multifaceted intervention to facilitate potential roll-out into routine care provision in Germany.

## Abbreviations

ADE: Adverse drug events
cRCT: Cluster-randomised controlled trial
GP(s): General practitioner(s)
MMSE: Mini-Mental State Examination
NHR: Nursing home resident(s)
PIM: Potentially inappropriate medications
